# The Secrets of Alternative Autophagy

**DOI:** 10.3390/cells10113241

**Published:** 2021-11-19

**Authors:** Kaja Urbańska, Arkadiusz Orzechowski

**Affiliations:** 1 Department of Morphological Sciences, Institute of Veterinary Medicine, Warsaw University of Life Sciences—SGGW, Nowoursynowska 159, 02-776 Warsaw, Poland; 2 Department of Physiological Sciences, Institute of Veterinary Medicine, Warsaw University of Life Sciences—SGGW, Nowoursynowska 159, 02-776 Warsaw, Poland

**Keywords:** alternative autophagy, ATG5, ATG7, Golgi apparatus, lysosome, RAB9, ULK1

## Abstract

For many years, it was thought that *ATG5* and *ATG7* played a pivotal role in autophagy, and that the knockdown of one of these genes would result in its inhibition. However, cells with *ATG5* or *ATG7* depletion still generate autophagic vacuoles with mainly *trans*-Golgi-originated isolation membranes and do not die. This indicates that autophagy can occur via ATG5/ATG7-independent alternative autophagy. Its molecular mechanism differs from that of the canonical pathway, including inter alia the phosphorylation of ULK1, and lack of LC3 modifications. As the alternative autophagy pathway has only recently been described, little is known of its precise role; however, a considerable body of evidence suggests that alternative autophagy participates in mitochondrion removal. This review summarizes the latest progress made in research on alternative autophagy and describes its possible molecular mechanism, roles and methods of detection, and possible modulators. There is a need for further research focused on types of autophagy, as this can elucidate the functioning of various cell types and the pathogenesis of human and animal diseases.

## 1. General Information about Autophagy

The term autophagy (from the Greek meaning “eating of self”) was first introduced by Christian de Duve in 1963 [1]. It refers to a phylogenetically ancient catabolic process taking place in eukaryotic cells, in which the cytosolic contents are trapped in double membranes, from where they are delivered to lysosomes and fused with them for degradation and recycling [2].

Of the three defined types of autophagy, macroautophagy is more prevalent than microautophagy and chaperone-mediated autophagy [3,4]. Macroautophagy, henceforth referred to as autophagy, is activated at critical times in response to cellular insults. These can range from lack of nutrients (lack of glucose or amino acids), energy (lack or insufficient ATP) or growth factors to oxidative or endoplasmic reticular stress, hypoxia, and pathogen invasion [3,5,6,7,8].

Given its biological significance, autophagy is regulated by a wide range of proteins and signaling pathways including mechanistic target of rapamycin (mTOR), phosphoinositide-3 kinase PI(3)K/AKT, mitogen-activated protein kinase (MAPK), AMP-activated protein kinase (AMPK) [9], cyclic AMP (cAMP)-activated protein kinase A [10], transcription factors [11], small GTPases, trimeric G proteins, inositol triphosphates, calcium signaling [9], and tumor suppressors [12]. In addition, DNA damage sensors [13], antiapoptotic proteins [14], and various hormones, growth factors, and reactive oxygen species (ROS) present in the cellular environment are important autophagy regulators [15].

Autophagy eliminates damaged, dysfunctional, or long-lived organelles (such as mitochondria or ribosomes). It also clears the cell from misfolded proteins, intracellular aggregates (i.e., glycans, lipids, and proteins) or simply excessive components [3,5,6,7,8]. The resulting “recycled” amino acids, fatty acids, nucleosides, nucleotides, and sugars are used to synthesize new macromolecules or regenerate metabolic precursors to produce ATP [7,16]. Thus, autophagy maintains metabolic homeostasis and ensures adaptation of the cell to changing environmental conditions [4]. It also participates in development, differentiation [17], immunity [4,18], senescence, and cell death [19]. Autophagy is also believed to play a dual role in tumor development and progression; while preventing or at least delaying initial tumor formation, it also appears to protect the malignant cells from environmental injury (i.e., during lack of nutrients, chemotherapy/radiotherapy) and support their progression once tumor formation has progressed [20]. Thus, autophagy inhibition increases the sensitivity of cancer cells to anticancer drugs. However, in contrast, autophagy overactivation (by pro-autophagic drugs) can also act as a cytotoxic mechanism and cause autophagic cell death, which suppresses tumor growth [21].

### Mechanism of Autophagy

The autophagic machinery consists of initiation, nucleation, elongation, maturation, fusion, and degradation steps [5,22]. Each is coordinated by autophagy-specific complexes, whose activity is strictly monitored by upstream signaling molecules [23].

In mammalian cells, in response to a signal for autophagy activation, endoplasmic reticulum (ER)-associated structures act as initiation sites and form a structure called an omegasome (the initiation stage). Following this, during nucleation, a membrane surrounds the cargo, expands, and forms a cup-shaped phagophore. The phagophore is a single-membrane sequestering compartment composed of the ER and the products obtained from the rupture of small membranous portions of the Golgi complex, endosomes, mitochondria, and plasma membrane. In the next phase, elongation, the phagophore continues to expand and engulf the cytoplasm with its content. In the maturation phase, complete closure of the phagophore leads to the formation of a spherical, double-membraned autophagosome. Following this, in the fusion phase, the autophagosome delivers the cargo to the lysosome, and the outer membrane belonging to the autophagosome becomes fused with the lysosomal membrane to form an autolysosome. Finally, degradation takes place, in which the hydrolases of the lysosome degrade the autophagosome inner membrane and the autophagic cargo, and the component parts are exported back into the cytoplasm through lysosomal permeases [2,5].

The initiation step is triggered by the activation of Unc-51-like autophagy activating kinase 1 (ULK1) or ULK2 complex composed of ULK1/2 itself, a FAK family interacting protein of 200 kDa (FIP200), and autophagy-related (ATG) 13 (ATG13) and 101 (ATG101). The autophagic activity of ULK1 can be regulated by phosphorylation at different sites [24,25]. During nutrient-rich conditions, the ULK1 complex is associated with the mTOR complex 1 (mTORC1), which phosphorylates ULK1 at Ser758 or Ser757, depending on the species. These events suppress ULK1 catalytic activity, which results in autophagy inhibition. Similarly, phosphorylation of ATG13 by mTORC1 negatively influences ULK1 activity and inhibits ULK1 complex relocation to the autophagy initiation sites. Lack of amino acids results in dephosphorylation of ULK1 and ATG13, which induces autophagy [2,26].

Cellular energy level also influences autophagy. A low ATP level or an increased AMP:ATP ratio activates AMPK. AMPK has a bidirectional influence on autophagy promotion: by inactivating mTORC1 or by phosphorylating ULK1 at different sites, leading to ULK1 activation. However, ATG13 may be phosphorylated by AMPK, resulting in autophagy inhibition [26]. Furthermore, triggering autophagy by ULK1 autophosphorylation at Thr180 is also possible [24]. ULK2 may compensate the loss of ULK1 [27].

Following activation, the ULK complex can relocate to the autophagy initiation sites and phosphorylate Beclin-1, thus activating the Beclin-1–VPS34–ATG14L–p150 complex and generating phosphatidylinositol 3-phosphate, a process crucial for the nucleation step. In the subsequent autophagy stages (elongation and maturation), microtubule-associated protein 1 light chain 3 (LC3) and ATG12 ubiquitin-like conjugation systems are involved [28].

ATG4 activates LC3 by cleaving the LC3 C-terminus, which generates cytosolic LC3-I [29]. All ATG4 isoforms (ATG4A, ATGB, ATG4C, ATG4D) participate in the priming of LC3, but mainly ATG4B participates in LC3 processing [30]. The LC3-I then conjugates to phosphatidylethanolamine (PE) via ATG7 and ATG3, where it transforms into insoluble LC3-II and is anchored to both the inner and the outer autophagosomal membranes. Unconjugated LC3-I is observed inside the cell [22]. ATG12 is conjugated with ATG5 by ATG7 and ATG10; this heterodimer interacts with ATG16L to allow the elongation and the maturation steps to take place [28,31].

The material intended for degradation is selected by targeted ubiquitination. The ubiquitination pattern is recognized by ubiquitin-interacting domains of autophagic cargo receptors: p62/SQSTM1 [28], Optineurin [32], NDP52 [33], NBR1, and TAX1BP1 [34]. These receptors, after binding with the ubiquitinated cargo [35], link with LC3 via the LC3-interacting regions. This binding facilitates targeting of the “marked” cargo to the autophagosome [28]. The fusion of the autophagosome to early/late endosomes or to lysosomes is controlled by the activity of Rab7, Lamps, Rubicon, SNAREs, and UVRAG [28]. LC3-I, which is localized on the outer membrane, is cleaved off by the cysteine protease ATG4 and recycled, while the LC3-II, localized on the inner membrane, is digested by lysosomal hydrolases together with the autophagolysosome content [36]. The products are then recycled back to the cytoplasm by lysosomal permeases [28,36].

## 2. Alternative Autophagy—A Short Overview

Both *ATG5* and *ATG7* are believed to be essential for autophagy [37]. Thus, the depletion of one of these genes was performed to inhibit this process [38].

However, cells lacking *ATG5* and/or *ATG7* still generate autophagic vacuoles [39] and are sensitive to treatment with the autophagy inhibitor chloroquine (CQ) [40]. The presence of phagophores and autophagosomes was confirmed in starved *Atg5^−/−^* mouse embryonic stem cells and *Atg5^−/−^* mouse embryonic fibroblasts (MEFs), as well as *Atg7^−/−^* hepatocytes and *Atg7^−/−^* MEFs [41]; however, they were small and were not generated as efficiently as in WT cells [42]. Indeed, ATG conjugation deficiency can delay autophagic activity [43], slowing the rate of autophagosome closure and the process of lysosomal fusion [44]. This suggests that *Atg5* and *Atg7* seem to be only partially involved in the particular steps of autophagy, and that the formation of autophagic isolation membranes is independent of the presence of an Atg conjugation system [41]. Indeed, canonical autophagy can also be performed in the absence of Atg5, but in a Syntaxin17-dependent manner [45]. Thus, in *ATG5* or *ATG7*-depleted cells, autophagy can progresses slowly [43,46] as residual canonical autophagy [43]. However, the ATG5/ATG7-independent autophagic pathway [29] called alternative autophagy [47] or, in yeast, the Golgi-membrane-associated degradation pathway (GOMED) [46] is also possible and could explain why *ATG5* or *ATG7* knockdown did not cause expected cell death [36,48,49,50,51].

The presence of the so-called ATG5/ATG7-independent, alternative autophagy pathway has been confirmed in a range of cells: fibroblasts and preadipocytes obtained from *Atg5*^flox/flox^ mice [48], knockout MEFs [45,52], thymocytes harvested from an *Atg5*^−/−^ mice embryo [47], mouse pancreatic β-cells [53], and induced pluripotent stem cells (iPSCs) [48]. Atg5-independent autophagy has also been described in cells from fetal brains, livers, and hearts [47], as well as in human epidermis [51]. In addition to normal cells, alternative autophagy has also been reported in various cancer cell lines, including prostate DU145 cancer cells [54,55], erythroleukemia K562 cells [6], adenocarcinoma H1650 cells, and lung carcinoma A549 cells [56].

### 2.1. Molecular Characteristic of Alternative Autophagy

The alternative autophagy pathway demonstrates similar morphological characteristics to canonical autophagy [45]. Both types are characterized by the formation of a range of typical autophagic structures, including autophagosomes, amphisomes, and autolysosomes, and the eventual digestion of engulfed material [47]. However, despite the formation of isolation membranes in cells deprived of an Atg conjugation system (including Atg3, Atg5, Atg7, Atg16L) [41], Atg12–Atg5 and LC3 conjugation systems seem to play a joint role in autophagosome maturation and closuring [57,58], as well as in the efficient degradation of its inner membrane [43]. Thus, the autophagosomes observed in cells lacking ATG5 or ATG7 could be generated by residual canonical autophagy. This may suggest the constant presence of canonical autophagy in cells with depleted *ATG5* and/or *ATG7*; canonical and alternative autophagy take place simultaneously, for example, during genotoxic stress conditions [46].

Although similar proteins regulate canonical and alternative autophagy processes at the initial phase, important differences exist between the types regarding the proteins involved in the further steps [54]. However, it could also be possible that proteins controlling the initial steps are required for canonical autophagy; however, ATG conjugation proteins are not listed among them [43].

The participation of PI(3)K (Beclin-1 and Vps34) and Ulk1 (Ulk1 and Fip200) complexes in an alternative autophagy pathway was confirmed by experiments using targeted inhibitors or by silencing their appropriate genes. Such manipulations suppressed autophagosome formation in *Atg5^−/−^* MEFs treated by different compounds or resulted in a decrease in the number of autophagic cells. Moreover, etoposide treatment resulted in the upregulation of Ulk1 in *Atg5*-depleted cells, which confirms the importance of Ulk1 in this pathway [47].

However, unlike canonical autophagy, this alternative autophagy pathway (provoked by genotoxic stress) is partially regulated by phosphorylation of Ulk1 at Ser746 (corresponding to Ser747 of ULK1), which takes place in the cytoplasm, shortly after autophagy induction. Following phosphorylation by receptor-interacting serine/threonine kinase 3 (RIPK3), the Ulk1 dissociates from the complex, and is transferred to the Golgi apparatus where it activates phagophore generation. RIPK3 transcription is regulated by TP53, and phosphorylation of Ulk1 at Ser746 is blocked in *p53* KO MEFs [52,59].

In addition, etoposide treatment results in Ulk1 dephosphorylation at Ser637 in a p53- and PPM1D (protein phosphatase, Mg^2+^/Mn^2+^-dependent 1D)-dependent manner. As this p53–PPM1D-assisted dephosphorylation is needed for Ulk1^Ser746^ phosphorylation, p53 appears to have a bidirectional effect on Ulk1 activity [52,59,60].

Moreover, studies based on etoposide-treated Atg5*^KO^*/Wipi3*^Cr^* MEFs and Atg5*^KO^* MEFs indicate that the induction of alternative autophagy requires Wipi3, but not Wipi2. Wipi3 is translocated from the cytoplasm to the *trans*-Golgi region, where it participates in the formation of isolation membranes and autophagic structures. This relocation of Wipi3 and the operation of the alternative autophagy pathway involve the activity of the Arg225 and Arg226 residues of Wipi3, as well as phosphatidylinositol 3-phosphate (PIP3). Although Wipi3 can also participates in canonical autophagy, its involvement in this autophagy pathway is minimal compared to Wipi2 [54].

As mentioned, isolation membranes mainly have *trans*-Golgi origins. In the expansion and closure, these membranes fuse with the endosome, resulting in the generation of autophagic vacuoles [39,46]. The participation of *trans*-Golgi and late endosomes in the formation of autophagic vacuoles was confirmed by fact that lysosomal protein 2 (Lamp-2)-positive autolysosomes colocalize with a fraction of mannose 6-phosphate receptors (*trans*-Golgi/late endosomes marker), TGN38 (the *trans*-Golgi marker) [47], and syntaxin 7 (late endosomes marker) [47], but not with calnexin (endoplasmic reticulum marker) [47]; in addition, a p-Ulk^Ser746^ signal was found to merge with a Golgi marker during immunofluorescence staining [59]. However, autophagic membranes can be generated from the endoplasmic reticulum (ER) in ATG5-depleted cells [44,58], and tubular structures participate in the conversion of the ER to isolation membrane [41].

After Ulk1 stimulation, the Rab9 protein, participating in late endosome–*trans*-Golgi trafficking, attaches to the autophagic membrane, thus allowing the generation of autophagosomes and autolysosomes [46]. GFP-Rab9 has also been found to colocalize with Lamp-2-positive autolysosomes, and Rab9 silencing is known to inhibit alternative autophagy but not the canonical one [47]. This indicates that alternative autophagy is Rab9-dependent [39,47]. Moreover, Rab9 overexpression inhibits the number of LC3-positive autophagosomes (via ATG4B upregulation) and promotes Rab9-mediated autophagosome generation. However, because of the involvement of other Rab proteins in the generation of autophagosomes during canonical autophagy [61], the role of Rab9 strictly limited to alternative autophagy needs to be further explored.

Moreover, in contrast to *Atg5* KO MEFs, etoposide treatment results in a lack of autophagosome and autolysosome formation in *Atg5/p53* double-KO MEFs, suggesting that the alternative autophagy induced by this genotoxic stressor is p53-dependent. The mechanism of p53 action involves the induction of damage-regulated autophagy modulator. Dram1 localizes on the Golgi membranes and participates in the process of elongation and closure of isolation membranes during etoposide-induced alternative autophagy. The loss of Dram1 inhibits autophagosome generation downstream of Ulk1. Although Dram1 may activate canonical autophagy in WT MEFs, its role is marginal in genotoxic stress-induced conventional autophagy, and Dram1 depletion does not act as a suppressor [45].

Most importantly, in contrast to WT MEFs, *Atg5^−^/^−^* MEFs do not show the presence of lipid conjugated LC3-II (in Western blot analysis) or a punctate pattern of green fluorescent protein (GFP)-tagged LC3 (under fluorescence microscopy). This lack of LC3 modification is a significant factor distinguishing alternative autophagy from canonical autophagy [47]. In *ATG7*-deficient cells, TAX1BP1 and TBK1 are required in the absence of LC3 lipidation. TAX1BP1 clusters with FIP200 around the NBR1-makred cargo, resulting in autophagosome formation [42]. However, in macrophages, the level of LC3–PE conjugation is dictated by their activation status and, unlike ATG5, ATG7 plays more of a supportive role [62]. A potential marker of alternative autophagy is the mentioned p-Ulk1^746^ [52].

The other molecule required for alternative autophagy is TRIM31, an intestine-specific protein localized in the mitochondria. TRIM31 facilitates autolysosome formation by interacting with mitochondrial PE in a palmitoylation-dependent manner. Although TRIM31 interacts with mitochondrial PE in both WT and Atg7^−/−^ cells, which activates autophagosome generation, TRIM31–PE binding induces an Atg5/Atg7-independent, alternative autophagy pathway. This autophagy can compensate for the function of canonical autophagy after the loss of Atgs [63].

**Figure 1:**
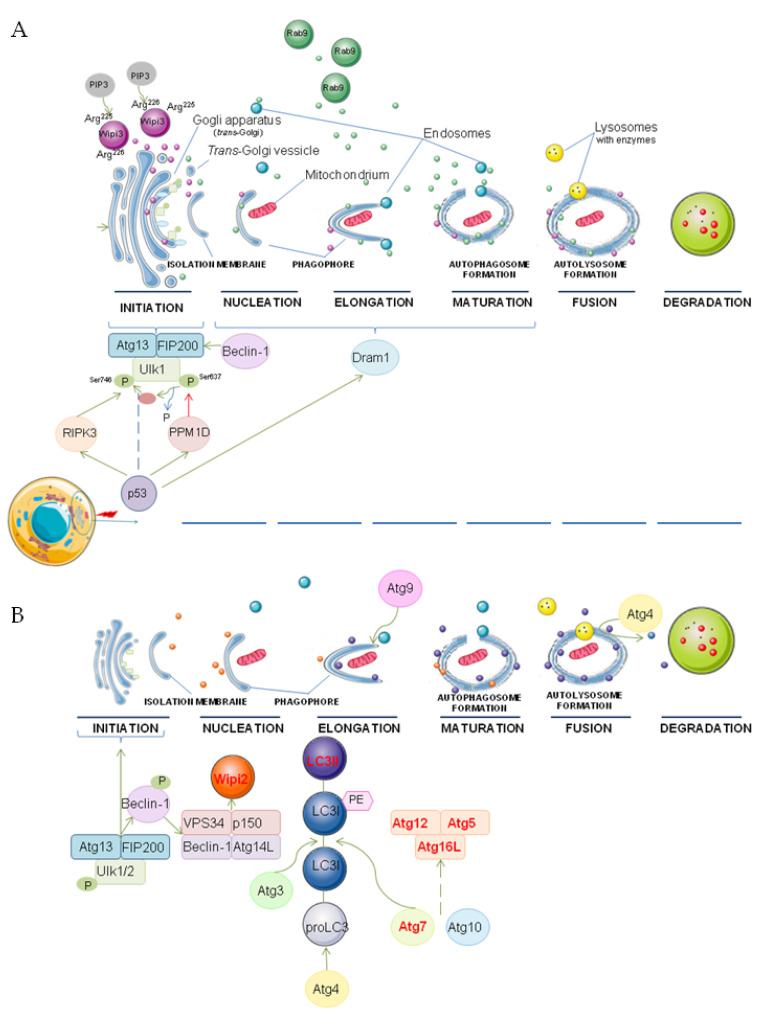
The possible molecular mechanism of genotoxic-induced alternative (**A**) and canonical (**B**) autophagy indicating proteins and organelles involved in these processes. P—phosphorylation, PE—phosphatidylethanolamine. The main figure conception was partially adapted from [47,52]. Graphical elements were adapted from Servier Medical Art (smart.servier.com accessed on 11 November 2021).

Depletion of *Atg7*, *Atg9*, *Atg12*, and *Atg16* was noted in *Atg5^−/−^* MEFs; however, this did not result in alternative autophagy inhibition under starvation conditions. This indicates that the pathway is independent of these genes and the proteins coded by them [47]. On the other hand, under basal, nutrient-rich conditions, ATG7-independent autophagy was described as ATG9A-dependent [42]. The possible molecular mechanism of genotoxic-induced alternative and canonical autophagy is illustrated in Figure 1.

### 2.2. Detection of Alternative Autophagy

Numerous techniques, mainly based on microscopy, can be used to monitor autophagy, measure autophagic flux, and identify the proteins involved in this process [45,47,52,54].

However, no universal marker of alternative autophagy exists, and this significantly impedes its detection and monitoring. It has been reported that no LC3 lipidation occurs during alternative autophagy [39]. Thus, the results based on LC3 conversion should be interpreted with caution [55]. Instead of LC3, Rab9, Syntaxin 7, and p-Ulk1^746^ (p-ULK1^747^) seem to be more appropriate potential markers [48,59].

Assuming that residual canonical autophagy does not occur in *ATG5-* and *ATG7-* depleted cells, for now, one of the most reliable techniques for confirming alternative autophagy in these cells is microscopy, particularly transmission electron microscopy (TEM). The presence of numerous autophagosomes, autolysosomes, or multilamellar bodies is a clear indicator of the autophagy process in both *Atg5*- [45,47] and *Atg7*-depleted mammalian cells [39]. Rapid freezing and freeze substitution should be performed during sample preparation to ensure good preservation of cellular components [47]. Even better results can be achieved using immunogold electron microscopy [63] or correlative light and electron microscopy (CLEM), which merges the photos from confocal fluorescence microscopy and TEM [52,54]. However, considering WT cells, it is not possible to use electron microscopy to distinguish structures formed during alternative and canonical autophagy, and this can lead to some misinterpretations. However, considering the essential role of ATG5 in the closure of isolation membranes, this misreading could also concern even *ATG5*-depleted cells, because opened autophagic structures (such as isolation membranes) are visible as sealed, round autophagosomes if cut tangentially [58]. The type of fixative used during sample preparation can also cause artefacts and influence the morphology of the autophagy structures under the microscope [41].

Although TEM is undoubtedly a valuable (in general) technique for monitoring autophagy, it is a time-consuming technique that requires a lot of experience and careful sample preparation [64]. An attractive alternative is fluorescence microscopy. Autolysosomes can be visualized using antibodies against Lamp-2 or Lamp-1 [45]. The number of cells containing Lamp-2-positive autolysosomes corresponds to the number of autophagic cells observed under TEM [45,47], with Lamp-2-positive dots indicating the presence of autolysosomes [47]. In non-autophagic cells, the lysosomes are seen as a small puncta [54] with a diffuse pattern [47]. During the autophagy process, due to the fusion of the lysosome with autophagic vacuoles, the autolysosomes are visible as large, ring-like [54] dots [45]. As such, an effective approach to monitoring this type of autophagy is the Lamp-2 swelling assay [52]. However, again, in WT cells, lysosome swelling can also occur due to the residual canonical autophagy. Thus, this assay does not discriminate between canonical and alternative autophagy.

Another tool suitable for studying alternative autophagy is the Keima-based assay. Keima, a fluorescence protein, indicates the presence of autolysosomes by emitting different-colored signals depending on pH (acidic vs. neutral). Alternatively, Cyto-ID dye can be used to selectively mark autophagic vacuoles; Cyto-ID puncta have been found to be similar to the autophagic vacuoles obtained by CLEM [45]. Autolysosome formation can also be detected using tandem fluorescence staining based on mRFP-GFP and mRFP-GFP-Rab9 proteins [54]. As GFP fluorescence decreases in acidic regions but RFP does not, autophagic cells possess a higher RFP/GFP fluorescence ratio and the autolysosomes are visible as red points [52]. In addition, the mCherry-Rab9 cleavage assay can also be used; however, lysates from mCherry-Rab9-expressing cells should be electrophoresed and then subjected to Western blot using anti-RFP antibody [54].

It is also possible to perform vital staining using acridine orange [65]—a cell-permeable green fluorophore which, after protonation, is trapped in acidic vacuolar organelles (AVOs) [66]. Cells containing AVOs can be observed by inverted fluorescence microscope [65,67]. Red fluorescence-emitting AVOs are visible as yellow, orange, or red granules, while non-AVOs are green; the resultant red-to-green fluorescence intensity ratio (R/G-FIR) can be used to calculate the R/GFIR index [65]. AVO formation can be also detected by flow cytometer (FL1 channel; excitation: 546 nm, emission: 575/640 nm) [67].

The classification of autophagic cells varies depending on the method used for quantification alternative autophagy. Some definitions require the cells to have autophagic vacuoles constituting more than 6% of the cytoplasmic region, and that at least 25 cells from one sample should be verified [45,47], while others define it as a cell which possesses more than one punctum (red puncta from mRFP-GFP, mCherry-puncta, or ring-like Lamp-2 puncta) with a diameter >1 µm [54] or ≥2 µm [52].

To predict autophagic cell death in vitro, three widely used colorimetric assays can be used: Crystal Violet (CV), 3-[4,5-dimethylthiaolyl]-2,5-diphenyl-tetrazolium bromide (MTT) reduction, and neutral red uptake (NR) assays. The results are expressed as autophagic arbitrary units (AAU), calculated according to the following formula:

$$\mathrm{AAU}=\left\{\frac{\mathrm{NR}}{[\mathrm{CV}+\mathrm{MTT}]/2}\right\}.$$

Although the results based on AAU are in agreement with LC3-II labeling [68], they do not discriminate between canonical and alternative autophagy. Similarly, residual canonical autophagy and alternative autophagy cannot be distinguished with autolysosome formation-dependent assays such as Keima, Cyto-ID, and acridine orange. The best methods for detecting and monitoring alternative autophagy seem to be CLEM, immunofluorescence, or Western blot, using antibodies binding specifically to the proteins involved in this pathway, if they are known.

### 2.3. The Modulators of Alternative Autophagy

Alternative autophagy can be activated upon starvation. Starvation-induced alternative autophagy was confirmed in Atg5^−/−^ MEFs [47] and Atg7^−/−^ erythroleukemia K562 cells [6]. On the contrary, lack of autophagosomes, but some autophagosome-like structures were observed in *Atg5*-deficient embryonic stem cells [69] and *Atg7*-deficient adult livers [70]. These different outcomes may result from differences in the peak times of the autophagic response after stimulus and duration of stress (6 h [47], 2 h [69], or 1 day [70]). However, the choice of cell type and autophagy detection method also seems to be important [29,45,47,70]. Moreover, autophagic response may be connected with differences in the signaling pathways, which use different *ATGs* [71]; as mentioned, ATG conjugation deficiency slows down autophagic activity [43]. Importantly, cells organized into tissues have higher sensitivity to losing autophagy competence due to their quiescence/post-mitotic state [72]. It can also explain the contradictory results obtained for starvation as an alternative autophagy trigger.

Alternative autophagy activation is also observed upon genotoxic stress [45,54] or hypoxia by disturbances in the Golgi-to plasma membrane pathway. These disturbances can also be achieved by silencing of the Golgi-localized PI(4) kinases PI4Kα and PI4Kβ [53] and the chemical compounds. Among the chemical inducers of alternative autophagy, the most common is etoposide, a DNA-damaging agent [6,45,47,54]. Etoposide causes the generation of autophagic vacuoles [37,47], including double-membraned autophagosomes and single-membraned autolysosomes [45,54], and it leads to an increase in the levels of Rab9A and Beclin-1 [6,45,47,54]. Camptothecin, another inductor of genotoxic stress, also triggers alternative autophagy, but via a different molecular mechanism than etoposide [45]. Staurosporine, a substance isolated from *Streptomyces staurosporeus* which acts as a nonspecific protein kinase inhibitor [73], was also successfully used to activate alternative autophagy [45,47,74] and generate Keima signals [45] or large Lamp-2-positive fluorescence dots [74]. Similarly, 1,3-cyclohexanebis (methylamine), known to inhibit anterograde trafficking by interfering with coatomer binding to Golgi membranes, also influences alternative autophagy activation, as confirmed by calculations based on ring-like Lamp-2 puncta [53,54].

The mTOR inhibitor rapamycin and mTOR-independent small-molecule enhancer of autophagy SMER28 [75] have also been used in studies related to alternative autophagy [48]. However, varying effects have been observed considering the effect of rapamycin; while rapamycin treatment was found to cause elevated mitophagy in *Atg5*^−/−^ tail-tip fibroblasts undergoing reprogramming [48], it was not able to activate autophagy in *Atg5*-deficinent mouse embryonic fibroblasts [37]. This could explain the observed failure of even high-dose rapamycin in breast cancer MCF-7 cell culture [76].

In addition, autolysosome formation is also observed after stimulation with lipopolysaccharide: an endotoxin isolated from Gram-negative bacteria [63]. Recent results also indicate that epibrassinolide, a polyhydroxysteroid similar to tetradrine isolated from *Stephania tetrandra* [55], also triggers Atg5/Atg7-independent autophagy [67], while carbonyl cyanide *m*-chlorophenylhydrazone (CCCP; a lipid-soluble acid) can be used to induce mitochondrion removal by alternative autophagy [6]. The compounds that can activate alternative autophagy are listed in Table 1.

Although autophagy can be inhibited by knocking down genes involved in the individual stages of process, pharmacological inhibition is more preferable, as it is more kinetically controllable [77]. The most commonly used inhibitor of alternative autophagy is Brefeldin A, a lactone than disrupts protein transport in the Golgi compartment [6,47,78].

Bafilomycin A1, a vacuolar-type H^+^ ATPase inhibitor, which causes autolysosome acidification and autophagosome–lysosome fusion failure [67], shifts the autophagosome-to-autolysosome ratio toward the former; in addition, the PI(3)K inhibitors, 3-methyladenine (3-MA) [47] and wortmannin, have been found to suppress autophagosome generation in etoposide-treated Atg5 deficient MEFs [74]. Lastly, CQ treatment, a weak base that inhibits lysosomal enzyme activity by raising the pH of the lysosome [77], did not cause ultrastructural differences in both *Atg5^+/+^* and *Atg5^−/−^* cells suggesting autophagy blockage also in *Atg5* knockout cells [79]. The compounds that can inhibit alternative autophagy are listed in Table 2.

However, the listed reagents (activators and inhibitors) can influence canonical autophagy. For example, although Brefeldin A was reported as a blocker of alternative autophagy which did not impact the canonical pathway [47], it has been found to impair canonical autophagy [78]. Thus, the employed reagents are dedicated to *ATG5*- or *ATG7*-depleted cells (assuming that *ATG5* or *ATG7* knockout indeed inhibits canonical autophagy efficiently). Otherwise, due to the lack of a selective method of detecting alternative autophagy, it is not possible to distinguish these two autophagic pathways, which are triggered by the same factor.

### 2.4. The Role of Alternative Autophagy

As it is a relatively new area of research and the knowledge regarding its characteristic proteins remains incomplete [39], relatively little is currently known about the biological roles of alternative autophagy.

However, it has been found that canonical and alternative autophagy pathways occur simultaneously in the same cell [29,53,63,80]; in addition, different signals can activate them [47], and they degrade different substrates, even after the same stimulus [39,59]. As such, the two pathways might have different functions [47]. It is also possible that alternative autophagy may serve as a compensatory mechanism in cells lacking *Atg5* or *Atg7* [6,63,81].

Alternative autophagy regulates many processes determining cell survival and the functioning of specific types of cells. For example, it mediates the proteolysis induced by genotoxic or nutrient stress. This may be the reason why 3-MA and Bafilomycin A inhibit protein degradation in starved or etoposide-treated *Atg5^−/−^* cells [47]. The process was found to enable the digestion of unused (pro)insulin granules released by β-cells cultured in glucose-deprived conditions; this would inhibit further insulin secretion in such conditions [53]. In addition, alternative autophagy participates in *Shigella* elimination and may act as a protective mechanism against bacteria-induced apoptosis [63].

Mutation in *ATG5* [82] or *ATG7* leads to neurodegeneration [83], because inhibited or inefficient autophagy results in the accumulation of ubiquitinated protein aggregates within neurons [84,85]. Although cells with loss of *ATG7* are regarded as “autophagy-deficient”, autophagosomes (some containing mitochondria) were present in muscle cells and fibroblasts derived from the patients with recessive variants in *ATG7.* This indicates that a lack of ATG does not completely stall canonical autophagy or that the observed autophagosomes were generated via alternative autophagy [83]. In this case, alternative autophagy (activated during chronic inhibition of canonical autophagy) could be a mechanism maintaining homeostasis [86]. Indeed, patients with biallelic deleterious *ATG7* variants have similar life expectancy to the general population [83]. However, this alternative autophagy may prompt further cellular damage (including nuclear breakdown) and lead to cell atrophy and finally degeneration [86]. Alternative autophagy may also be responsible for ensuring correct axis projection in growing nerve cells. If this is the case, any failures in the pathway may underpin the development of neurodegenerative disorders such as Alzheimer’s disease, dentatorubral–pallidoluysian atrophy, and Niemann–Pick disease type C [23,46]. Importantly, Rab9 controls the type of autophagy pathway in the pancreas and participates in canonical to Rab9-dependent, alternative autophagy switching. This switch also aggravates pancreatitis. Thus, canonical and alternative autophagy may act antagonistically [61].

Some studies have noted the impact of *ATG5* and *ATG7* depletion on mitochondria biology and cellular energetic status, but in the context of total autophagy inhibition [38,87]; however, their findings are sometimes contradictory. In the case of cells lacking *ATG5* or *ATG7*, autophagic flux estimation should not be based solely only on LC3 conversion [88], as this is not affected in alternative autophagy [55]; in such cases, the analysis should be supplemented with methods dedicated to alternative autophagy.

In tumor-derived cell lines, *Atg7* depletion did not cause mitochondrial dysfunction, because the number of nonsynonymous mutations to trigger such an effect was too low; however, *Atg7* deficiency did result in the reduction of metabolite recycling during starvation, particularly regarding TCA cycle intermediates including glutamate, aspartate, and α-ketoglutarate. This indicates that substrate limitation in these tumor cells impaired mitochondrial metabolism [87]. In addition, *ATG7* knockout PANC-1 cells demonstrated elevated levels of glutamine, glutamate, and aspirate during glycolysis suppression [89]. Silencing *ATG7* was also found to decrease glucose uptake and lactate secretion in chronic myeloid leukemia cells; in this case, the cells generated ATP through oxidative phosphorylation (OXPHOS), which facilitated ROS-dependent differentiation [90]. *ATG5* silencing impaired OXPHOS and reduced mitochondrial function, but similar glycolysis rates and glycolytic capacity, reflected by the extracellular acidification rate, were observed between primary *Atg5* KO and *Atg5* WT tumor cells [38]. In contrast, *ATG5* or *ATG7* deficiency in Bel7402 and SMMC7721 human liver cancer cells resulted in increased glycolytic activity, measured by glucose consumption and lactate production [88]. These results suggest that, depending on the cell type, alternative autophagy (or eventually residual canonical autophagy) mediates the regulation of glucose metabolism and metabolic reprogramming observed in many types of cancer.

Although it is difficult to unequivocally determine the role of *ATG5* and *ATG7* in cellular respiration, a considerable body of evidence suggests that alternative autophagy is associated with mitochondrion removal—mitophagy. Indeed, in a genetic knockout mouse model, the lack of *Atg7* only resulted in a delayed clearance of mitochondria in reticulocytes, while normal mitochondrial clearance was observed in cells derived from *Atg5* knockout mice [91], suggesting the existence of an alternative mitophagy process [74]. Moreover, in erythroleukemia K562 cells, both canonical and alternative mitophagic mechanisms were observed; if canonical autophagy was dysfunctional, the alternative mitophagy was still capable of effectively removing damaged or excessive mitochondria [6]. In reticulocytes, the mitochondria clearance taking place via Atg5-independent alternative autophagy facilitated fetal erythrocyte differentiation. More importantly, alternative mitophagy was described as a major pathway for mitochondria removal in fetal definitive reticulocytes compared to primitive and adult definitive cells [6,47,74].

The same mitophagy pathway was found to participate in the reprogramming process of iPSCs [48]; in contrast, Atg7-independent mitophagy regulated ROS levels and DNA damage repair (via regulation of RAD50) and suppressed apoptosis in erythroleukemia cells exposed to radiation or CCCP [6]. Moreover, Atg7-independent mitophagy was described as the main form of mitochondrial clearance in the heart tissue during starvation conditions and protected cardiomyocytes against ischemic injury [92]. Although *ATG7* depletion did not influence mitophagy in HeLa cells, this process was effectively inhibited by *RAB9* depletion [80].

**Figure 2:**
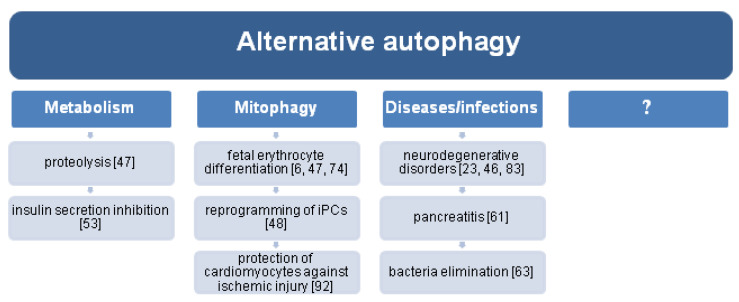
The roles of alternative autophagy. Graphical elements were adapted from Servier Medical Art (smart.servier.com).

More precisely, alternative mitophagy is mediated through the Ulk1/Rab9/Rip1 (receptor-interacting serine/threonine protein kinase 1)/Drp1 (dynamin-related protein 1) axis. Energy stress, such as ischemia, triggers phosphorylation of Rab9^Ser179^ by ULK1, which facilitates Rab9–Rip1 association and Drp1^Ser616^ phosphorylation. The activation of Drp1 induces of mitochondrial fission and engulfment by the double-membrane compartment of the *trans*-Golgi membrane [92]. The activity of MAPK1/ERK2 and MAPK14/p38 signaling pathways is also crucial for alternative mitophagy, induced by starvation or hypoxia [80]. A brief overview of the roles of alternative autophagy is illustrated in Figure 2.

## 3. Conclusive Remarks

A considerable body of evidence suggests that alternative autophagy is essential for proper cell development and survival, but it can also act bidirectionally with regard to disease development. A detailed understanding of the importance of both ATG5 and ATG7 in various biological processes, as well as the precise role and molecular mechanism of ATG5/ATG7-independent alternative autophagy, can elucidate the functioning of many cell types and the pathogenesis of various illnesses (including neurogenerative, metabolic, and neoplastic diseases). The results gained from such studies may lead to new therapeutic strategies in the prevention or treatment of various diseases. Bearing in mind the role of mitophagy in various aspects of normal and cancer cell biology, alternative mitophagy seems to be an interesting area of research, and targeting mitophagy could bring some benefits, depending on the context. However, considering the fine line between residual canonical autophagy and alternative autophagy, all results based on *ATG5* and *ATG7* depletion need to be carefully studied and interpreted.

## Figures and Tables

**Table 1:** Alternative autophagy activators.

Alternative Autophagy Activators			
Name of Drug	Concentration	Time of Treatment	Cell Type
Etoposide	10 µM [37,45,47,54] 20 µM [6]	18 h [37,45,47,54] 18 h [6]	*Atg7* KO K652 leukemia cells [6], Atg5/p53 DKO MEFs [45], *Atg5* KO MEFs [45,47,54]
Camptothecin [45]	10 µM	12 or 18 h	*Atg5* KO MEFs [45]
Staurosporine	1 µM [45,47,74]	12 h [45,47] or 24 h [74]	*Atg5* KO MEFs [45,47], *Atg5* KO thymocytes [47], *Atg5* KO erythroid cells [74]
1,3-cyclohexanebis (methylamine) [53]	2 mM	3, 6, or 24 h	*Atg5* KO MEFs, Atg5 KO MIN6 insulinoma cells
Rapamycin [37,48] *	0.3 nM [48]	6–8 h each day for the first 6 days [48] **	*Atg5* KO mice tail-tip fibroblasts (confirmed) [48], *Atg5* KO MEFs (not confirmed) [37]
SMER28 [48]	1 µM		*Atg5* KO mice tail-tip fibroblasts
Lipopolysaccharide [63]	100 ng·mL^−1^	3, 4, or 10 h	*Atg5* KO MEFs
Epibrassinolide [67]	30 μM	24–48 h	*Atg5* KO MEFs
Carbonyl cyanide *m*-chlorophenylhydrazone [6]	20 µM	18 h	*Atg7* KO K652 erythroleukemia cells

* Contradictory data; ** drug used in reprogramming.

**Table 2:** Alternative autophagy inhibitors.

Alternative Autophagy Inhibitors			
Name of Drug	Concentration	Time of Treatment	Cell Type
Brefeldin A	0.1 μg/mL [6,47,78]	18 h [47]	*Atg7* KO K652 erythroleukemia cells [6], *Atg5* KO MEFs [47]
Bafilomycin A1 [47]	10 nM	18 h	*Atg5* KO MEFs
3-Methyladenine [47]	5 mM	18 h	*Atg5* KO MEFs
Wortmannin [74]	1 µM	From 3 to 6 days of cell incubation	Reticulocytes from *Atg5* KO mouse embryo
Chloroquine [79]	10–25 µM	no data	*Atg5* KO M9 leukemia cells

## References

[1] Klionsky D.J. (2008). Autophagy Revisited: A Conversation with Christian de Duve. Autophagy.

[2] Parzych K.R., Klionsky D.J. (2014). An Overview of Autophagy: Morphology, Mechanism, and Regulation. Antioxid. Redox Signal..

[3] Glick D., Barth S., Macleod K.F. (2010). Autophagy: Cellular and Molecular Mechanisms. J. Pathol..

[4] Feng Y., He D., Yao Z., Klionsky D.J. (2014). The Machinery of Macroautophagy. Cell. Res..

[5] Li X., He S., Ma B. (2020). Autophagy and Autophagy-Related Proteins in Cancer. Mol. Cancer.

[6] Wang J., Fang Y., Yan L., Yuan N., Zhang S., Xu L., Nie M., Zhang X., Wang J. (2016). Erythroleukemia Cells Acquire an Alternative Mitophagy Capability. Sci. Rep..

[7] Roca-Agujetas V., de Dios C., Lestón L., Marí M., Morales A., Colell A. (2019). Recent Insights into the Mitochondrial Role in Autophagy and Its Regulation by Oxidative Stress. Oxid. Med. Cell. Longev..

[8] Chun Y., Kim J. (2018). Autophagy: An Essential Degradation Program for Cellular Homeostasis and Life. Cells.

[9] Thorburn A. (2008). Apoptosis and Autophagy: Regulatory Connections between Two Supposedly Different Processes. Apoptosis.

[10] Grisan F., Iannucci L.F., Surdo N.C., Gerbino A., Zanin S., Di Benedetto G., Pozzan T., Lefkimmiatis K. (2021). PKA Compartmentalization Links CAMP Signaling and Autophagy. Cell Death Differ..

[11] Song T.-T., Cai R.-S., Hu R., Xu Y.-S., Qi B.-N., Xiong Y.-A. (2021). The Important Role of TFEB in Autophagy-Lysosomal Pathway and Autophagy-Related Diseases: A Systematic Review. Eur. Rev. Med. Pharmacol. Sci..

[12] Sutton M.N., Huang G.Y., Liang X., Sharma R., Reger A.S., Mao W., Pang L., Rask P.J., Lee K., Gray J.P. (2019). DIRAS3-Derived Peptide Inhibits Autophagy in Ovarian Cancer Cells by Binding to Beclin1. Cancers.

[13] Cordani M., Donadelli M., Strippoli R., Bazhin A.V., Sánchez-Álvarez M. (2019). Interplay between ROS and Autophagy in Cancer and Aging: From Molecular Mechanisms to Novel Therapeutic Approaches. Oxid. Med. Cell. Longev..

[14] Cheung C.H.A., Chang Y.-C., Lin T.-Y., Cheng S.M., Leung E. (2020). Anti-Apoptotic Proteins in the Autophagic World: An Update on Functions of XIAP, Survivin, and BRUCE. J. Biomed. Sci..

[15] Gonzalez C.D., Alvarez S., Ropolo A., Rosenzvit C., Gonzalez Bagnes M.F., Vaccaro M.I. (2014). Autophagy, Warburg, and Warburg Reverse Effects in Human Cancer. BioMed Res. Int..

[16] Galluzzi L., Pietrocola F., Bravo-San Pedro J.M., Amaravadi R.K., Baehrecke E.H., Cecconi F., Codogno P., Debnath J., Gewirtz D.A., Karantza V. (2015). Autophagy in Malignant Transformation and Cancer Progression. EMBO J..

[17] Mizushima N., Levine B. (2010). Autophagy in Mammalian Development and Differentiation. Nat. Cell. Biol..

[18] Crotzer V.L., Blum J.S. (2009). Autophagy and Its Role in MHC-Mediated Antigen Presentation. J. Immunol..

[19] Yoshii S.R., Mizushima N. (2017). Monitoring and Measuring Autophagy. Int. J. Mol. Sci..

[20] Mathew R., Karantza-Wadsworth V., White E. (2007). Role of Autophagy in Cancer. Nat. Rev. Cancer.

[21] Li F., Guo H., Yang Y., Feng M., Liu B., Ren X., Zhou H. (2019). Autophagy Modulation in Bladder Cancer Development and Treatment. Oncol. Rep..

[22] Codogno P., Mehrpour M., Proikas-Cezanne T. (2012). Canonical and Non-Canonical Autophagy: Variations on a Common Theme of Self-Eating?. Nat. Rev. Mol. Cell. Biol..

[23] Park H., Kang J.-H., Lee S. (2020). Autophagy in Neurodegenerative Diseases: A Hunter for Aggregates. Int. J. Mol. Sci..

[24] Hurley J.H., Young L.N. (2017). Mechanisms of Autophagy Initiation. Annu. Rev. Biochem..

[25] Zachari M., Longo M., Ganley I.G. (2020). Aberrant Autophagosome Formation Occurs upon Small Molecule Inhibition of ULK1 Kinase Activity. Life Sci. Alliance.

[26] Zachari M., Ganley I.G. (2017). The Mammalian ULK1 Complex and Autophagy Initiation. Essays Biochem..

[27] Lee E.-J., Tournier C. (2011). The Requirement of Uncoordinated 51-like Kinase 1 (ULK1) and ULK2 in the Regulation of Autophagy. Autophagy.

[28] Sun K., Deng W., Zhang S., Cai N., Jiao S., Song J., Wei L. (2013). Paradoxical Roles of Autophagy in Different Stages of Tumorigenesis: Protector for Normal or Cancer Cells. Cell Biosci..

[29] Juenemann K., Reits E.A. (2012). Alternative Macroautophagic Pathways. Int. J. Cell Biol..

[30] Agrotis A., Pengo N., Burden J.J., Ketteler R. (2019). Redundancy of Human ATG4 Protease Isoforms in Autophagy and LC3/GABARAP Processing Revealed in Cells. Autophagy.

[31] Kaufmann A., Beier V., Franquelim H.G., Wollert T. (2014). Molecular Mechanism of Autophagic Membrane-Scaffold Assembly and Disassembly. Cell.

[32] Bansal M., Moharir S.C., Swarup G. (2018). Autophagy Receptor Optineurin Promotes Autophagosome Formation by Potentiating LC3-II Production and Phagophore Maturation. Commun. Integr. Biol..

[33] Thurston T.L.M., Ryzhakov G., Bloor S., von Muhlinen N., Randow F. (2009). The TBK1 Adaptor and Autophagy Receptor NDP52 Restricts the Proliferation of Ubiquitin-Coated Bacteria. Nat. Immunol..

[34] Turco E., Savova A., Gere F., Ferrari L., Romanov J., Schuschnig M., Martens S. (2021). Reconstitution Defines the Roles of P62, NBR1 and TAX1BP1 in Ubiquitin Condensate Formation and Autophagy Initiation. Nat. Commun..

[35] Padman B.S., Nguyen T.N., Uoselis L., Skulsuppaisarn M., Nguyen L.K., Lazarou M. (2019). LC3/GABARAPs Drive Ubiquitin-Independent Recruitment of Optineurin and NDP52 to Amplify Mitophagy. Nat. Commun..

[36] Komatsu M., Ichimura Y. (2010). Physiological Significance of Selective Degradation of P62 by Autophagy. FEBS Lett..

[37] Arakawa S., Honda S., Yamaguchi H., Shimizu S. (2017). Molecular Mechanisms and Physiological Roles of Atg5/Atg7-Independent Alternative Autophagy. Proc. Jpn. Acad. Ser. B Phys. Biol. Sci..

[38] Lin H.H., Chung Y., Cheng C.-T., Ouyang C., Fu Y., Kuo C.-Y., Chi K.K., Sadeghi M., Chu P., Kung H.-J. (2018). Autophagic Reliance Promotes Metabolic Reprogramming in Oncogenic KRAS-Driven Tumorigenesis. Autophagy.

[39] Shimizu S. (2018). Biological Roles of Alternative Autophagy. Mol. Cells.

[40] Gallagher L.E., Radhi O.A., Abdullah M.O., McCluskey A.G., Boyd M., Chan E.Y.W. (2017). Lysosomotropism Depends on Glucose: A Chloroquine Resistance Mechanism. Cell Death Dis..

[41] Uemura T., Yamamoto M., Kametaka A., Sou Y., Yabashi A., Yamada A., Annoh H., Kametaka S., Komatsu M., Waguri S. (2014). A Cluster of Thin Tubular Structures Mediates Transformation of the Endoplasmic Reticulum to Autophagic Isolation Membrane. Mol. Cell. Biol..

[42] Ohnstad A.E., Delgado J.M., North B.J., Nasa I., Kettenbach A.N., Schultz S.W., Shoemaker C.J. (2020). Receptor-Mediated Clustering of FIP200 Bypasses the Role of LC3 Lipidation in Autophagy. EMBO J..

[43] Tsuboyama K., Koyama-Honda I., Sakamaki Y., Koike M., Morishita H., Mizushima N. (2016). The ATG Conjugation Systems Are Important for Degradation of the Inner Autophagosomal Membrane. Science.

[44] An H., Harper J.W. (2018). Systematic Analysis of Ribophagy in Human Cells Reveals Bystander Flux during Selective Autophagy. Nat. Cell. Biol..

[45] Nagata M., Arakawa S., Yamaguchi H., Torii S., Endo H., Tsujioka M., Honda S., Nishida Y., Konishi A., Shimizu S. (2018). Dram1 Regulates DNA Damage-Induced Alternative Autophagy. Cell Stress..

[46] Honda S., Arakawa S., Yamaguchi H., Torii S., Tajima Sakurai H., Tsujioka M., Murohashi M., Shimizu S. (2020). Association Between Atg5-Independent Alternative Autophagy and Neurodegenerative Diseases. J. Mol. Biol..

[47] Nishida Y., Arakawa S., Fujitani K., Yamaguchi H., Mizuta T., Kanaseki T., Komatsu M., Otsu K., Tsujimoto Y., Shimizu S. (2009). Discovery of Atg5/Atg7-Independent Alternative Macroautophagy. Nature.

[48] Ma T., Li J., Xu Y., Yu C., Xu T., Wang H., Liu K., Cao N., Nie B., Zhu S. (2015). Atg5-Independent Autophagy Regulates Mitochondrial Clearance and Is Essential for IPSC Reprogramming. Nat. Cell. Biol..

[49] Fukuda T., Oda K., Wada-Hiraike O., Sone K., Inaba K., Ikeda Y., Makii C., Miyasaka A., Kashiyama T., Tanikawa M. (2016). Autophagy Inhibition Augments Resveratrol-Induced Apoptosis in Ishikawa Endometrial Cancer Cells. Oncol. Lett..

[50] Kuma A., Hatano M., Matsui M., Yamamoto A., Nakaya H., Yoshimori T., Ohsumi Y., Tokuhisa T., Mizushima N. (2004). The Role of Autophagy during the Early Neonatal Starvation Period. Nature.

[51] Li L., Chen X., Gu H. (2016). The Signaling Involved in Autophagy Machinery in Keratinocytes and Therapeutic Approaches for Skin Diseases. Oncotarget.

[52] Torii S., Yamaguchi H., Nakanishi A., Arakawa S., Honda S., Moriwaki K., Nakano H., Shimizu S. (2020). Identification of a Phosphorylation Site on Ulk1 Required for Genotoxic Stress-Induced Alternative Autophagy. Nat. Commun..

[53] Yamaguchi H., Arakawa S., Kanaseki T., Miyatsuka T., Fujitani Y., Watada H., Tsujimoto Y., Shimizu S. (2016). Golgi Membrane-associated Degradation Pathway in Yeast and Mammals. EMBO J..

[54] Yamaguchi H., Honda S., Torii S., Shimizu K., Katoh K., Miyake K., Miyake N., Fujikake N., Sakurai H.T., Arakawa S. (2020). Wipi3 Is Essential for Alternative Autophagy and Its Loss Causes Neurodegeneration. Nat. Commun..

[55] Qiu W., Zhang A.-L., Tian Y. (2017). Tetrandrine Triggers an Alternative Autophagy in DU145 Cells. Oncol. Lett..

[56] Zhang P., Ling L., Zheng Z., Zhang Y., Wang R., Wu M., Zhang N., Hu M., Yang X. (2021). ATG7-Dependent and Independent Autophagy Determine the Type of Treatment in Lung Cancer. Pharmacol. Res..

[57] Sou Y., Waguri S., Iwata J., Ueno T., Fujimura T., Hara T., Sawada N., Yamada A., Mizushima N., Uchiyama Y. (2008). The Atg8 Conjugation System Is Indispensable for Proper Development of Autophagic Isolation Membranes in Mice. Mol. Biol. Cell..

[58] Kishi-Itakura C., Koyama-Honda I., Itakura E., Mizushima N. (2014). Ultrastructural Analysis of Autophagosome Organization Using Mammalian Autophagy-Deficient Cells. J. Cell. Sci..

[59] Wen X., Klionsky D.J. (2020). Phosphorylation of ULK1 Serine 746 Dictates ATG5-Independent Autophagy. Autophagy.

[60] Torii S., Yoshida T., Arakawa S., Honda S., Nakanishi A., Shimizu S. (2016). Identification of PPM1D as an Essential Ulk1 Phosphatase for Genotoxic Stress-Induced Autophagy. EMBO Rep..

[61] Mareninova O.A., Dillon D.L., Wightman C.J.M., Yakubov I., Takahashi T., Gaisano H.Y., Munson K., Ohmuraya M., Dawson D., Gukovsky I. (2021). Rab9 Mediates Pancreatic Autophagy Switch From Canonical to Noncanonical, Aggravating Experimental Pancreatitis. Cell. Mol. Gastroenterol. Hepatol..

[62] Vujić N., Bradić I., Goeritzer M., Kuentzel K.B., Rainer S., Kratky D., Radović B. (2021). ATG7 Is Dispensable for LC3-PE Conjugation in Thioglycolate-Elicited Mouse Peritoneal Macrophages. Autophagy.

[63] Ra E.A., Lee T.A., Won Kim S., Park A., Choi H.J., Jang I., Kang S., Hee Cheon J., Cho J.W., Eun Lee J. (2016). TRIM31 Promotes Atg5/Atg7-Independent Autophagy in Intestinal Cells. Nat. Commun..

[64] Fodor E., Sigmond T., Ari E., Lengyel K., Takács-Vellai K., Varga M., Vellai T. (2017). Methods to Study Autophagy in Zebrafish. Methods Enzymol.

[65] Jaśkiewicz A., Pająk B., Litwiniuk A., Urbańska K., Orzechowski A. (2018). Geranylgeraniol Prevents Statin-Dependent Myotoxicity in C2C12 Muscle Cells through RAP1 GTPase Prenylation and Cytoprotective Autophagy. Oxid. Med. Cell. Longev..

[66] Thomé M.P., Filippi-Chiela E.C., Villodre E.S., Migliavaca C.B., Onzi G.R., Felipe K.B., Lenz G. (2016). Ratiometric Analysis of Acridine Orange Staining in the Study of Acidic Organelles and Autophagy. J. Cell. Sci..

[67] Adacan K., Obakan-Yerlikaya P., Arisan E.D., Coker-Gurkan A., Kaya R.I., Palavan-Unsal N. (2020). Epibrassinolide-Induced Autophagy Occurs in an Atg5-Independent Manner Due to Endoplasmic Stress Induction in MEF Cells. Amino Acids.

[68] Martins W.K., Severino D., Souza C., Stolf B.S., Baptista M.S. (2013). Rapid Screening of Potential Autophagic Inductor Agents Using Mammalian Cell Lines. Biotechnol. J..

[69] Mizushima N., Yamamoto A., Hatano M., Kobayashi Y., Kabeya Y., Suzuki K., Tokuhisa T., Ohsumi Y., Yoshimori T. (2001). Dissection of Autophagosome Formation Using Apg5-Deficient Mouse Embryonic Stem Cells. J. Cell. Biol..

[70] Komatsu M., Waguri S., Ueno T., Iwata J., Murata S., Tanida I., Ezaki J., Mizushima N., Ohsumi Y., Uchiyama Y. (2005). Impairment of Starvation-Induced and Constitutive Autophagy in Atg7-Deficient Mice. J. Cell. Biol..

[71] Zhu W., Qu H., Xu K., Jia B., Li H., Du Y., Liu G., Wei H.-J., Zhao H.-Y. (2017). Differences in the Starvation-Induced Autophagy Response in MDA-MB-231 and MCF-7 Breast Cancer Cells. Anim. Cells Syst..

[72] Klionsky D.J., Petroni G., Amaravadi R.K., Baehrecke E.H., Ballabio A., Boya P., Bravo-San Pedro J.M., Cadwell K., Cecconi F., Choi A.M.K. (2021). Autophagy in Major Human Diseases. EMBO J..

[73] Ding Y., Wang B., Chen X., Zhou Y., Ge J. (2017). Staurosporine Suppresses Survival of HepG2 Cancer Cells through Omi/HtrA2-Mediated Inhibition of PI3K/Akt Signaling Pathway. Tumour Biol..

[74] Honda S., Arakawa S., Nishida Y., Yamaguchi H., Ishii E., Shimizu S. (2014). Ulk1-Mediated Atg5-Independent Macroautophagy Mediates Elimination of Mitochondria from Embryonic Reticulocytes. Nat. Commun..

[75] Koukourakis M.I., Giatromanolaki A., Fylaktakidou K., Sivridis E., Zois C.E., Kalamida D., Mitrakas A., Pouliliou S., Karagounis I.V., Simopoulos K. (2018). SMER28 Is a MTOR-Independent Small Molecule Enhancer of Autophagy That Protects Mouse Bone Marrow and Liver against Radiotherapy. Invest. New Drugs.

[76] Yellen P., Saqcena M., Salloum D., Feng J., Preda A., Xu L., Rodrik-Outmezguine V., Foster D.A. (2011). High-Dose Rapamycin Induces Apoptosis in Human Cancer Cells by Dissociating MTOR Complex 1 and Suppressing Phosphorylation of 4E-BP1. Cell Cycle.

[77] Mauthe M., Orhon I., Rocchi C., Zhou X., Luhr M., Hijlkema K.-J., Coppes R.P., Engedal N., Mari M., Reggiori F. (2018). Chloroquine Inhibits Autophagic Flux by Decreasing Autophagosome-Lysosome Fusion. Autophagy.

[78] Crowley L.C., O’Donovan T.R., Nyhan M.J., McKenna S.L. (2013). Pharmacological Agents with Inherent Anti-Autophagic Activity Improve the Cytotoxicity of Imatinib. Oncol. Rep..

[79] Chen X., Clark J., Guan J., Kumar A.R., Zheng Y. (2015). Susceptibility of AML to Chloroquine Therapy Is Independent of Autophagy. Blood.

[80] Hirota Y., Yamashita S., Kurihara Y., Jin X., Aihara M., Saigusa T., Kang D., Kanki T. (2015). Mitophagy Is Primarily Due to Alternative Autophagy and Requires the MAPK1 and MAPK14 Signaling Pathways. Autophagy.

[81] Ljubojević-Holzer S., Kraler S., Djalinac N., Abdellatif M., Voglhuber J., Schipke J., Schmidt M., Kling K.-M., Franke G.T., Herbst V. (2021). Loss of Autophagy Protein ATG5 Impairs Cardiac Capacity in Mice and Humans through Diminishing Mitochondrial Abundance and Disrupting Ca^2+^ Cycling. Cardiovasc. Res..

[82] Kim M., Sandford E., Gatica D., Qiu Y., Liu X., Zheng Y., Schulman B.A., Xu J., Semple I., Ro S.-H. (2016). Mutation in ATG5 Reduces Autophagy and Leads to Ataxia with Developmental Delay. Elife.

[83] Collier J.J., Guissart C., Oláhová M., Sasorith S., Piron-Prunier F., Suomi F., Zhang D., Martinez-Lopez N., Leboucq N., Bahr A. (2021). Developmental Consequences of Defective ATG7-Mediated Autophagy in Humans. N. Engl. J. Med..

[84] Nah J., Yuan J., Jung Y.-K. (2015). Autophagy in Neurodegenerative Diseases: From Mechanism to Therapeutic Approach. Mol. Cells.

[85] Nixon R.A. (2013). The Role of Autophagy in Neurodegenerative Disease. Nat. Med..

[86] Baron O., Boudi A., Dias C., Schilling M., Nölle A., Vizcay-Barrena G., Rattray I., Jungbluth H., Scheper W., Fleck R.A. (2017). Stall in Canonical Autophagy-Lysosome Pathways Prompts Nucleophagy-Based Nuclear Breakdown in Neurodegeneration. Curr. Biol..

[87] Guo J.Y., Teng X., Laddha S.V., Ma S., Van Nostrand S.C., Yang Y., Khor S., Chan C.S., Rabinowitz J.D., White E. (2016). Autophagy Provides Metabolic Substrates to Maintain Energy Charge and Nucleotide Pools in Ras-Driven Lung Cancer Cells. Genes Dev..

[88] Jiao L., Zhang H.-L., Li D.-D., Yang K.-L., Tang J., Li X., Ji J., Yu Y., Wu R.-Y., Ravichandran S. (2018). Regulation of Glycolytic Metabolism by Autophagy in Liver Cancer Involves Selective Autophagic Degradation of HK2 (Hexokinase 2). Autophagy.

[89] Shiratori R., Furuichi K., Yamaguchi M., Miyazaki N., Aoki H., Chibana H., Ito K., Aoki S. (2019). Glycolytic Suppression Dramatically Changes the Intracellular Metabolic Profile of Multiple Cancer Cell Lines in a Mitochondrial Metabolism-Dependent Manner. Sci. Rep..

[90] Karvela M., Baquero P., Kuntz E.M., Mukhopadhyay A., Mitchell R., Allan E.K., Chan E., Kranc K.R., Calabretta B., Salomoni P. (2016). ATG7 Regulates Energy Metabolism, Differentiation and Survival of Philadelphia-Chromosome-Positive Cells. Autophagy.

[91] Ding W.-X., Yin X.-M. (2012). Mitophagy: Mechanisms, Pathophysiological Roles, and Analysis. Biol. Chem..

[92] Saito T., Nah J., Oka S.-I., Mukai R., Monden Y., Maejima Y., Ikeda Y., Sciarretta S., Liu T., Li H. (2019). An Alternative Mitophagy Pathway Mediated by Rab9 Protects the Heart against Ischemia. J. Clin. Investig..

